# Prognostic significance of FDG-PET/CT based radiomics analysis in newly-diagnosed multiple myeloma: a comparative study with clinical assessment

**DOI:** 10.3389/fonc.2025.1486495

**Published:** 2025-09-03

**Authors:** Fei Li, Baiyang Jiang, Ye Fu, Qingyang Yu, Guangwen Duan, Jiayang Yan, Qinling Jiang, Hongbiao Sun, Yi Xiao, Qi Chen, Shaochun Xu, Xiang Wang, Shiyuan Liu

**Affiliations:** ^1^ Department of Research and Development, Shanghai United Imaging Intelligence Co., Ltd., Shanghai, China; ^2^ Department of Radiology, Changzheng Hospital, Naval Medical University, Shanghai, China; ^3^ Department of Radiology, Kunshan Third People’s Hospital, Kunshan, Jiangsu, China

**Keywords:** multiple myeloma, radiomics, 18-FDG PET/CT, computer-aided diagnosis, prognostic value

## Abstract

**Objective:**

This study aimed to construct and validate a fusion diagnostic model based on Fluorodeoxyglucose-Positron Emission Tomography/Computed Tomography(FDG-PET/CT) radiomics for predicting overall survival of multiple myeloma (MM) patients.

**Methods:**

A total of 199 patients newly diagnosed with MM were included from two centers. All patients underwent whole-body PET**/**CT scans within one month before the initiation of treatment and were followed up for over five years. Radiomic features of MM were extracted from CT images and dimensionality reduction was performed by LASSO regression analysis. Cox Proportional Hazards Model was then constructed to predict patient survival. A clinical-radiomic fusion model was constructed by integrating independent clinical risk factors, including comprehensive laboratory parameters, R-ISS, and PET functional metabolic parameters, with the radiomic model. The discrimination ability of the model was evaluated using the C-index, and it’s calibration was assessed using calibration curves.

**Results:**

The C-indexes for the radiomics model in the training and testing cohorts were 0.736 and 0.708, respectively; for the clinical model, they were 0.676 and 0.696, respectively; and for the integrated model, they were 0.791 and 0.776, respectively. The integrated diagnostic model outperformed both the radiomics and clinical models, showcasing higher discriminative ability and improved calibration. In the training set, the C-index was 0.791 (95% confidence interval [CI]: 0.713-0.853), with an ICI of 0.015, E50 of 0.014, and AIC of 10.987. In the testing set, the C-index was 0.776 (95% CI: 0.654–0.894), with an ICI of 0.069, E50 of 0.04, and AIC of 11.492.

**Conclusions:**

This integrated prediction model exhibited satisfactory performance in predicting survival outcomes for patients diagnosed with MM and improved precision in discriminating between patients with a good prognosis and poor prognosis.

## Introduction

Multiple myeloma (MM) is a hematological malignancy, which is characterized by the abnormal production of monoclonal immunoglobulin M components within plasma cells situated in the bone marrow (1). Worldwide, MM is responsible for about 1% of cancer deaths, mainly affecting people aged 65–70 and more common in males, accounting for 10-15% of hematological malignancies (2). Significant progress has been made in treatment strategies, particularly with the development of new drugs that can elicit long-lasting responses. However, the prognosis for specific patients has shown limited improvement (3). A critical aspect of clinical practice involves accurately assessing patient risks and implementing precise treatment protocols. Osteopathy in MM patients significantly affect their quality of life and increase both incidence rates and mortality (4–6). Imaging examination plays a critical role in managing MM patients. According to the latest staging system, accurate treatment selection, follow-up procedures, and prognosis evaluation for newly diagnosed patients are significantly dependent on imaging assessments such as CT, Magnetic Resonance Imaging (MRI), or PET/CT.

CT, MRI, and PET/CT imaging modalities are increasingly utilized in clinical practice for their ability to offer a thorough evaluation of tumor burden. Additionally, these imaging methods offer supplementary predictive information for patients receiving systemic treatment. Whole-body low-dose computed tomography (WBLDCT) is a highly accurate method for detecting osteolytic lesions while offering a low radiation dose (7, 8). The introduction of whole-body MRI has redirected attention in imaging towards bone marrow and extramedullary involvement (9). However, variations in signal intensity within bone marrow involvement present notable differences linked to age disparities (10). The International Myeloma Working Group (IMWG) recommends FDG-PET as a valuable technique for visualizing and offering functional and metabolic information of lesions, thereby highlighting disease activity. This method is particularly useful in monitoring treatment response, especially in cases of oligo-secretory and non-secretory myeloma, and aid in disease monitoring for patients with these particular subtypes.

PET/CT modality plays a vital role in the initial staging, treatment assessment, and minimal residual disease determination in MM (11, 12). However, its primarily utilization is dependent on subjective visual assessment by clinicians and basic quantitative metrics like maximum standardized uptake value (SUVmax) for evaluating MM tumor burden and predicting prognosis (13). Bartel et al. demonstrated that baseline MRI and PET/CT scans have prognostic significance for event-free survival and overall survival (OS) in patients (14). In clinical practice, quantitative values such as SUVmax, metabolic tumor volume (MTV), and total lesion glycolysis(TLG) primarily reflect the metabolic functional information of MM lesions, while neglecting a more in-depth analysis of CT anatomical information. Therefore, it is important to study the quantitative features of CT images to enhance predictive accuracy in MM by analyzing the spatial distribution and metabolic heterogeneity of focal lesions in MM.

Radiomics entails the extraction of lesion imaging features through high-throughput analysis, yielding quantitative parameters that are frequently undetectable by visual inspection (15–17). The increasing interest among researchers in radiomic research highlights its significance as a valuable tool for evaluating the prognosis of tumor patients. Nevertheless, numerous radiomic models fail to incorporate clinical and laboratory data. We hypothesize that by integrating baseline PET/CT radiomics information with genetic and laboratory examination indicators, personalized prognostic evaluation models can be constructed. In this retrospective multi-center study, we developed a prediction model for multiple myeloma (MM) using a combination of multi-modal PET/CT radiomics data and various clinical variables from two medical centers.

## Materials and methods

### Study population

A total of 199 patients diagnosed with MM were included in this study between December 2015 and December 2022. Each patient was diagnosed as MM through comprehensive histological and hematological examinations, adhering to the IMWG guidelines for both diagnosis and treatment (18). Additionally, all patients provided written informed consent to participate. Full-body PET/CT examinations were conducted within one month before the initiation of treatment for each patient.

The exclusion criteria were: (a) patients with combined malignant tumors or hematological diseases in other systems; (b) patients with concomitant cardiac amyloidosis; (c) patients received chemotherapy and radiation therapy before the examination; (d) patients with poor image quality. Following the inclusion criteria, a total of 199 patients (110 males and 89 females) were enrolled, with a median age of 61 years and an age range between 34 and 86 years. All patients underwent comprehensive blood-based laboratory tests. Laboratory indicators encompassed M protein, sFLC (free light chain), hemoglobin, creatinine, albumin, Ca2+, lactate dehydrogenase, β2-microglobulin levels, platelets, hypersensitive C-reactive protein, and PET/CT quantitative parameters. Serum protein, serum albumin, glucose filtration rate, beta-2 microglobulin, hemoglobin, hematocrit, calcium levels, and serum lactate dehydrogenase were additionally quantified. The treatment plans are divided into three categories: chemotherapy regimens based on proteasome inhibitor–based therapy(PI-based), chemotherapy regimens based on immunomodulatory drug-based therapy(IMiD-based), and chemotherapy regimens based on the combination of proteasome inhibitor-containing therapy and immunomodulatory drug-based therapy(IMiD+PI).

**Figure 1:**
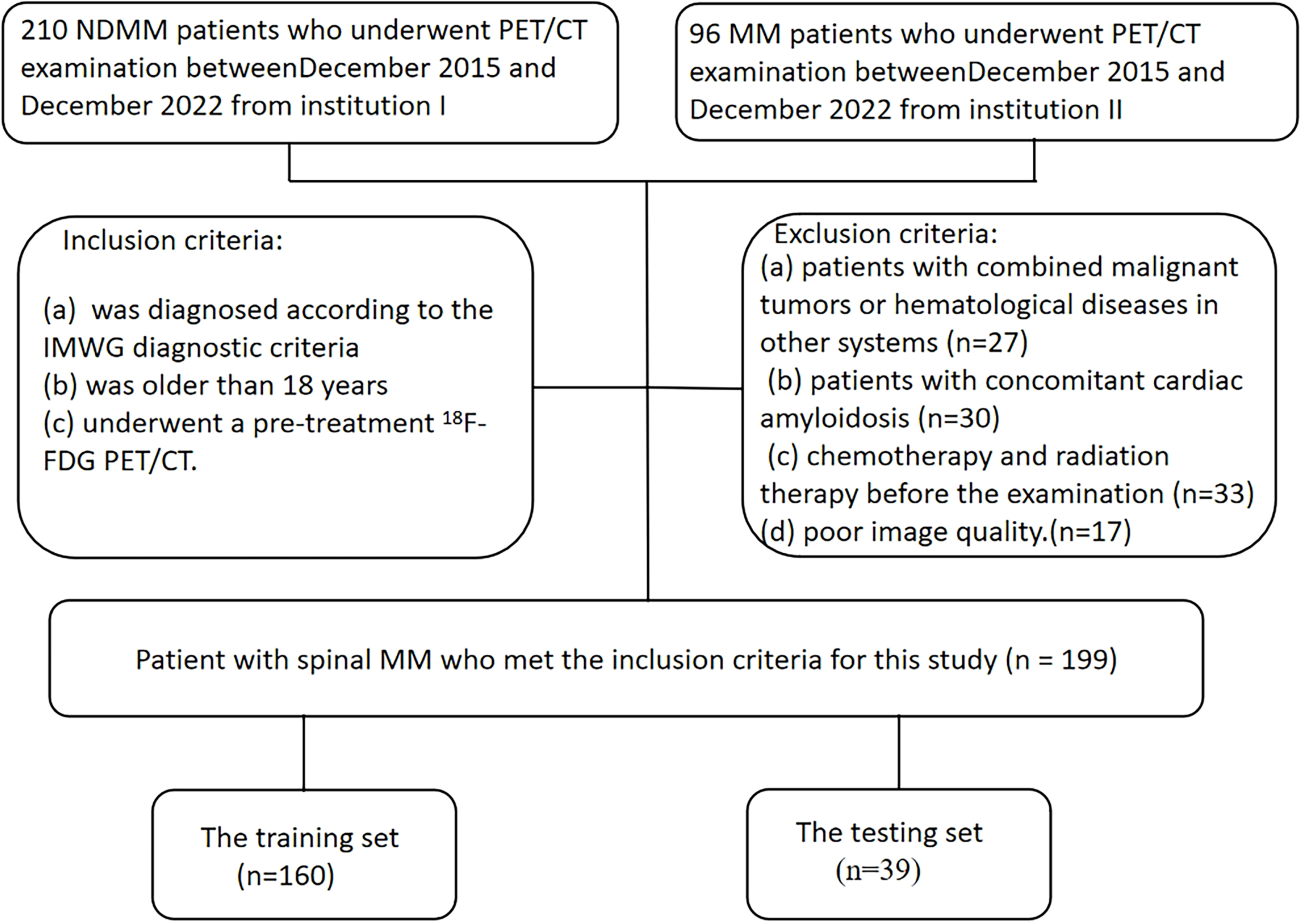
Flowchart summarizing patient enrolment process and study cohorts.

Imaging data for this study were obtained from two centers, with clinical treatment administered at Changzheng Hospital for a cohort of 199 patients. The primary endpoint of the study was overall survival (OS), defined as the duration from disease diagnosis to death or last follow-up. OS was calculated from the time of initial diagnosis until death or the study’s endpoint in December 2022 for surviving patients. The baseline characteristics and treatment information of patients are described in **Table 1**. A flowchart illustrating patients selection and the study process is presented in **Figure 1**.

**Table 1:** Basic patient information of training set and testing set.

Variables	Training set (n=160)	Testing set (n=39)	P-values
Age (mean±sd)	59.681 ± 9.531	60.077 ± 8.585	0.801
Gender (0/1)			
Male	19	91	0.358
Female	20	69	
Initial treatment plan			
PI-based	115	27	0.773
IMiD-based	11	4	
1IMiD+PI	34	8	
Bone marrow plasma cell (≥60%)			
Yes	29	7	0.980
No	131	32	
FL (≥3)			
Yes	131	34	0.430
No	29	5	
EMD			
Yes	75	16	0.511
No	85	23	
HB (≤100g/L)			
Yes	77	19	0.947
No	83	20	
Cr (≥177umol/L)			
Yes	22	7	0.772
No	138	32	
ALB (≥35g/L)			
Yes	74	22	0.255
No	86	17	
LDH (≥250U/L)			
Yes	22	7	0.505
No	138	32	
β2-MG (≥5.5mg/L)			
Yes	44	10	0.814
No	116	29	
PLT (<100*10^9/L)			
Yes	16	4	0.056
No	144	35	
HCRP (>10mg/L)			
Yes	39	7	0.280
No	121	32	
Ca2. (≥2.55mmol/L)			
Yes	22	5	0.112
No	138	34	
DS.staging (1/2/3)			
I	11	4	0.207
II	36	4	
III	113	31	
ISS.staging			
I	22	10	0.677
II	60	12	
III	67	17	
RISS.staging (1/2/3)			
I	27	9	0.290
II	100	19	
III	33	11	
liver (Median[Q1~Q3])	2.07[1.8~2.423]	2.17[1.805~2.395]	0.921
SUVmax (Median[Q1~Q3])	5.525[4.085~7.633]	5.65[4.365~8.08]	0.485
TLG (Median[Q1~Q3])	41[17.5~79.5]	39[17.5~83]	0.920
MTV (Median[Q1~Q3])	12[5~24]	12[5.5~30]	0.856

FL, focal lesion; EMD, extramedullary; MTV, metabolic tumor volume; TLG, total lesion glycolysis; Cr, creatinine; ALB, albumin; LDH, lactate dehydrogenase; β 2M, β 2-microglobulin levels; PLT, platelet; HCRP, hypersensitive C-reactive protein; R-ISS, the revised International Staging System; DS staging, Durie Salmon staging; SUVmax, max standardized uptake value; PI-based, Proteasome inhibitor-based; IMiD-based, immunomodulatory drug-based.

### Equipment and parameters

All images were obtained from the hospital’s Picture Archiving and Communication System (PACS) and scanned using the SIEMENS Biograph 64-layer PET/CT equipment. Patients underwent a fasting period of over 6 hours before being injected with 18F-FDG at a concentration of 0.15-0.18 mCi/kg. Typically, the PET/CT scan commenced 60 minutes post-injection. Patients were positioned supine with both upper arms placed above their heads to minimize chest artifacts.

The procedure began with a body CT scan, using scanning parameters of tube voltage of 120 kV, tube current of 150 mA, layer thickness of 3mm, and scanning range from the top of the skull to the middle of the femur. The body PET/CT scan was collected for 5–6 beds, with a conventional collection time of two minutes per bed. Subsequently, CT data was utilized for attenuation correction and PET image enhancement, followed by image reconstruction and fusion. Lesions were further analyzed via multi-planar reconstruction at the post-processing workstation.

### PET image delineation and registration between PET and CT images

Two experienced radiologists, with 6 and 7 years of expertise respectively, conducted blind segmentation of lesions exhibiting the highest uptake on PET scans. In cases of discrepancies concerning PET/CT findings, a consensus was reached through a collaborative review involving a senior nuclear medicine physician with over 10 years of experience. The interpretation of PET images adhered to IMWG standards, defining focal lesions as those exhibiting higher uptake than the hematopoietic bone marrow background (BM) or liver, with a minimum diameter of 5 mm. Diffuse uptake was defined as uptake above that of the liver (19).

Evaluation of images was carried out by a team of experienced nuclear medicine physicians (W.X. and X.S.) following established criteria for assessing myeloma lesions. Briefly, positive areas were indicated by the presence of focal areas with increased tracer uptake within bones (SUV ≥2.5), with or without any underlying lesions identified on CT or osteolytic CT areas >0.5 cm (20). The total Metabolic Tumor Volume (TMTV) was defined as the volume of all MM lesions throughout the body on PET/CT with an SUV≥2.5. To enhance segmentation consistency, two radiologists randomly selected 20 patients for intra- and inter-observer consistency tests. Intra-group and inter-group consistency coefficients (ICC) between features were computed to identify and retain features demonstrating robust repeatability (ICC>0.70).

**Figure 2:**
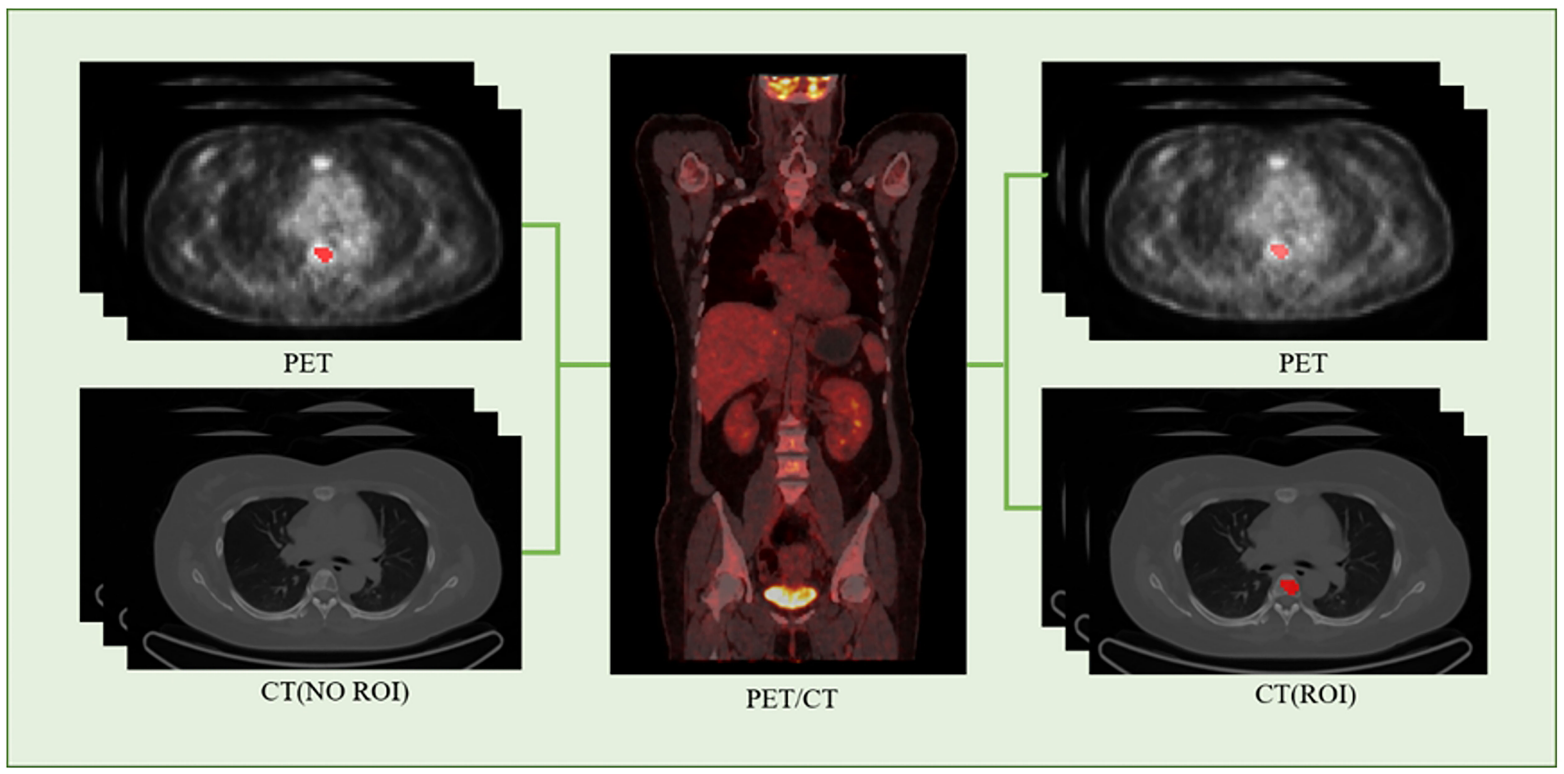
PET-CT registration process flowchart.

We employed the PET/CT registration method available on the platform (https://www.uii-ai.com/en/uai/scientific-research) for the automated alignment of PET and CT images. Subsequently, a senior medical radiologist reviewed the registered images to confirm the precise alignment of major organ boundaries, such as the skin, skeletal structures, and liver. A registration matrix was generated to quantify this alignment. Ultimately, the regions of interest (ROI) identified from the PET images were overlaid onto corresponding locations within CT images (**Figure 2**).

### Radiomics extraction and selection

**Figure 3:**
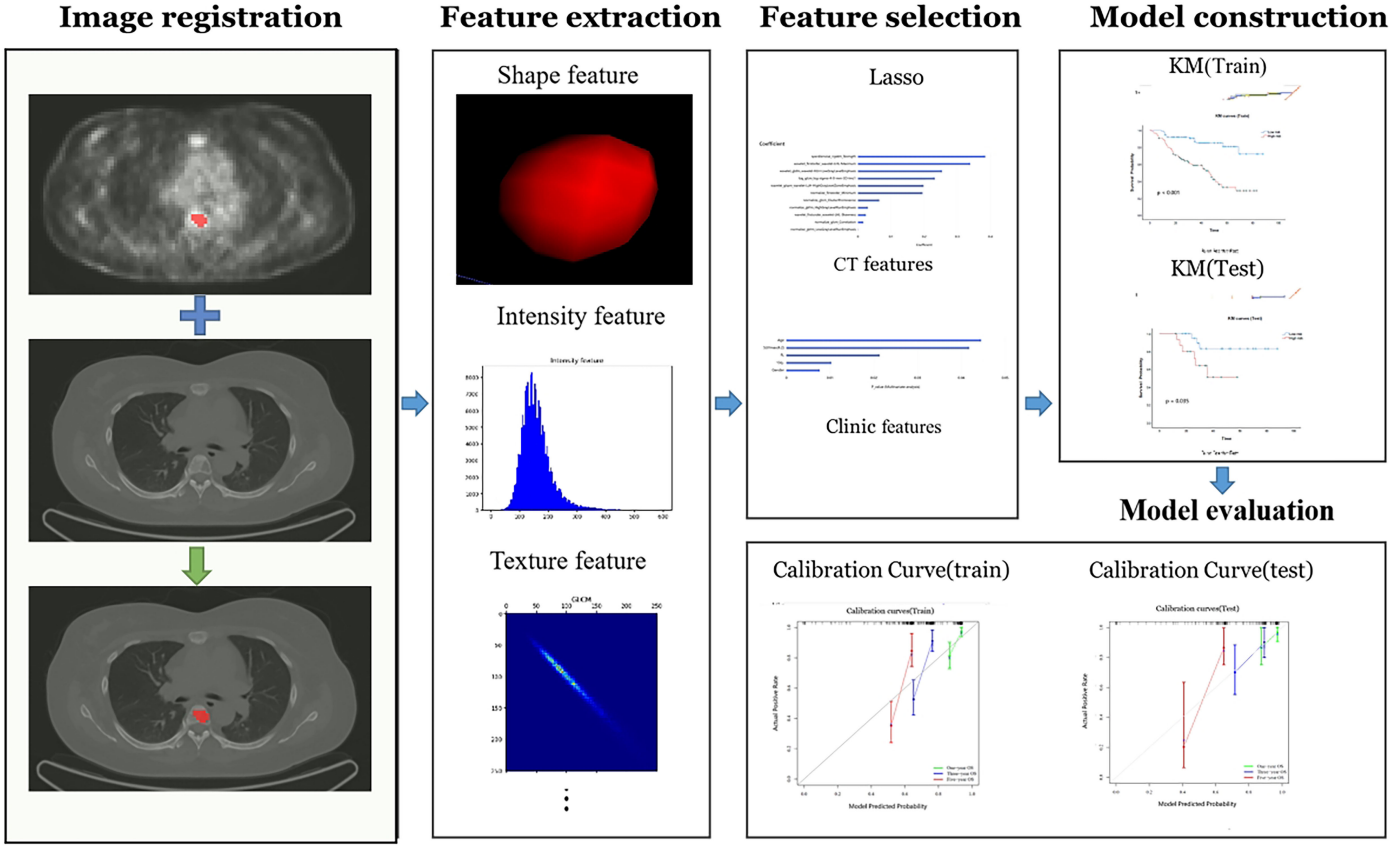
The radiomics workchart by uAI research portal. On the medical images, segmentation is performed to define the fibroids region. From this region the features are extracted, e.g. fibroids shape, intensity, and texture features. And then these features are used for analysis, which Those important features will be selected and used to construct predictive model. Finally, the model is evaluated.

The flowchart of the radiomics analysis is shown in **Figure 3**. Initially, all parametric maps underwent normalization using maximum and minimum truncation processing. Subsequently, 14 image filters were used to generate derived images, from which first-order statistics and texture features were extracted, resulting in a total of 2,160 derived features. From the largest focal area of myeloma in each patient, 2,264 radiomics features were automatically extracted. These features encompass three groups: 14 shape features, 450 first-order features quantifying the distribution of voxel intensities in images, and 1,800 texture features. The texture features consist of 525 gray level co-occurrence matrix (GLCM) features, 350 gray level run length matrix (GLRLM) features, 400 gray level size zone matrix (GLSZM) features, 400 neighboring gray tone difference matrix (NGTDM) features, 125 gray level dependent matrix (GLDM) features, collectively capturing regional heterogeneity differences. All radiomics features were standardized using Z-score normalization to mitigate dimensional disparities.

For PET data, the functional metabolic parameters SUVmax, MTV, and TLG are indicators for indicating the tumor metabolic information. CT images are more suitable for radiomics extraction tasks related to texture, shape, and intensity features of multiple myeloma. We employed the Least Absolute Shrinkage and Selection Operator (LASSO) selection method to identify the most reliable predictive radiomic features. Initially, we focused on radiomics features extracted from CT images, leveraging these extracted features to construct a Cox proportional hazards regression model. Then we merged these CT features with clinical and PET features (SUVmax, MTV and TLG) to obtain a comprehensive combined model.

### Prognosis model

#### Predictive task

Our predictive task aimed to accurately forecast the prognosis of MM patients. To mitigate risks of bias and overfitting, we employed two methods. Firstly, we filtered features by employing the intraclass correlation coefficient (ICC) within and between observers, establishing a threshold of ICC > 0.70. Secondly, we applied the least absolute shrinkage and selection operator (LASSO) to the training dataset, using a five-fold cross-validation approach to identify the most predictive features. These strategies were employed to identify the most informative features while ensuring optimal predictive performance.

#### Development and validation of the predictive model

We developed three models: the Clinical Model, the CT Radiomics Model, and the Combined Model. In the Clinical Model, we conducted a univariate analysis (p < 0.1) using COX regression on clinical data, encompassing parameters such as SUVmax, MTV, and TLG PET quantitative parameters. Moreover, based on the clinical relevance and results of the univariate Cox analysis (p < 0.05), candidate factors potentially associated with OS were identified. These candidate factors were further incorporated into a multivariable Cox regression model, facilitating the selection of clinical variables that could impact OS and contributing to the construction of the clinical model.

Regarding the CT radiomics model, we employed LASSO to select radiomics features extracted from the images. These selected features were subsequently employed in a COX regression analysis to construct the radiomics model. In the Combined Model, we integrated clinical risk factors and radiomics features to establish a combined model. This combined model was used to further predict postoperative survival in MM patients. To assess the efficacy of our model, we evaluated the performance of the CT Radiomics Model, Clinical Model, and Combined Model based on both discrimination and calibration.

To assess discrimination, we compared these models using four metrics: C-index values, Integrated Calibration Index (ICI), E50 statistic, and Akaike Information Criterion (AIC). The Akaike Information Criterion (AIC) is a widely used measure for evaluating the goodness-of-fit and complexity of a model. It is calculated as AIC = 2k - 2ln(L), where k represents the number parameters of in the model, and L is the maximum likelihood estimate of the model. In this study, we employed the Cox proportional hazards model to calculate the likelihood values for each model and subsequently used AIC to compare the performance of different models. The C-index is another important metric used to evaluate the predictive ability of a survival model, measuring the consistency between the survival probabilities predicted by the model and the actual survival outcomes. The calculation of the C-index is based on all possible patient pairs, assessing agreement by comparing the predicted survival times with the actual survival times. Specifically, the C-index is calculated as the ratio of the number of consistent pairs to the number of useful pairs. In our analysis, we utilized the survival and survcomp packages in R to compute the C-index and evaluate the predictive ability of the model separately in the training set and the test set.

These metrics provided a comprehensive evaluation of each model’s efficacy in distinguishing between patients. Additionally, calibration curves were used to evaluate the calibration of each model, enabling us to pinpoint models with superior calibration performance. In summary, through comprehensive evaluation from both discrimination and calibration perspectives, we identified the optimal predictive model among the CT Radiomics Model, Clinical Model, and Combined Model for predicting postoperative survival in patients with MM.

### Statistical analysis

To assess the normality of continuous features, we employed the Kolmogorov-Smirnov test. The T-test was used to compare variables with a normal distribution, which are represented as mean ± SD (standard deviation). For non-normally distributed data, the Mann-Whitney U test was used, and the data was represented using the median (inter-quartile range). Categorical variables were analyzed using either the chi-square test or Fisher’s exact test. The data was represented as counts (%). Survival analysis was conducted via Kaplan-Meier survival curves and validated using the log-rank test. A p-value lower than 0.05 was considered statistically significant. The R software package (version 4.0.3) was used to process the demographic data for evaluating significant differences in the variables between the training and the validation set. Python (version 3.6) was employed for programming model training, validating the prediction model, as well as conducting statistical analysis.

## Results

### Basic characteristics

This study included a total of 199 patients (110 males: with a median age of 61 years). Patients were continuously monitored until either the date of demise or August 1, 2022, with a median follow-up period of 35 months for the entire cohort. Among them, 64 patients survived. The treatment plan involved 160 patients within the training set and 39 patients within the testing set.

### Assessment of radiomic features

**Figure 4:**
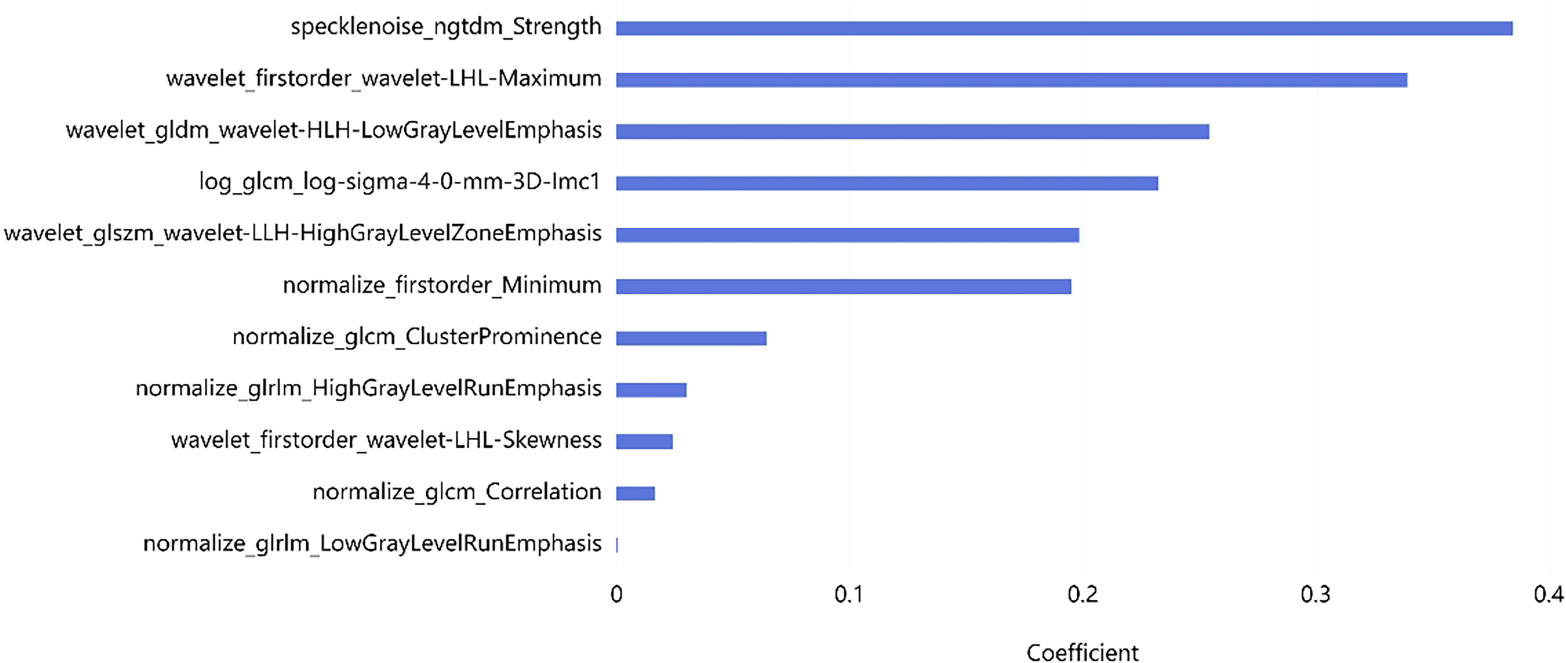
Radiomics feature selection. The 11 most significant feature subsets selected by LASSO.

A total of 2,264 radiomics features were initially extracted from CT images, with a specific emphasis on the largest lesions of MM. Subsequent refinement through consistency testing yielded 2,151 robust features. To select the most relevant features, we employed the LASSO algorithm in conjunction with five-fold cross-validation. Features that received three or more votes in a voting process were retained. Following this rigorous selection process, a subset of 11 optimal radiomic features was identified for implementation in machine learning models. This subset comprised three first-order features, and eight texture features (**Figure 4**). The coefficient of each selected feature is shown in **Table 2**.

**Table 2:** Results of radiomic feature selection for OS.

Feature name	Coefficient
specklenoise_ngtdm_Strength	0.454943
wavelet_firstorder_wavelet-LHL-Maximum	0.413943
wavelet_gldm_wavelet-HLH-LowGrayLevelEmphasis	0.281109
log_glcm_log-sigma-4-0-mm-3D-Imc1	0.278916
normalize_firstorder_Minimum	0.233923
wavelet_glszm_wavelet-LLH-HighGrayLevelZoneEmphasis	0.230632
normalize_glcm_ClusterProminence	0.085334
normalize_glrlm_HighGrayLevelRunEmphasis	0.042087
wavelet_firstorder_wavelet-LHL-Skewness	0.031114
normalize_glcm_Correlation	0.009674
normalize_glrlm_LowGrayLevelRunEmphasis	0.001089

### Assessment of clinic features

Nine clinical features were extracted following univariate analysis, with significant correlations observed with OS for HCRP, SUVmax, ISS-staging, RISS-staging, PLT, Age, FL, Bone Marrow Plasma Cell, and Gender (p<0.10). Subsequent adjustment for relevant factors using the Cox multivariate model revealed that Age, SUVmax, FL, and Gender were identified da independent predictors of OS in the clinical setting (**Table 3**).

**Table 3:** Univariate and multivariate Cox regression analysis.

Parameters	Univariate analysis (p value)	Multivariate analysis (p value)
Age	0.03	0.04
Gender	0.00	0.00
TLG	0.89	
ALB	0.75	
EMD	0.71	
MTV	0.54	
HB	0.43	
liver	0.42	
LDH	0.31	
Ca2+	0.30	
b2M	0.22	
Cr	0.16	
DS-staging	0.13	
HCRP	0.09	
SUVmax	0.06	0.04
ISS-staging	0.05	
RISS-staging	0.04	
PLT	0.03	
FL	0.01	0.02
BoneMarrowPlasmaCell	0.00	

### Comparison between different models

A radiomics model and a clinical model were constructed by utilizing radiomic features selected through LASSO screening and clinical features chosen through Cox multivariate analysis, respectively. Furthermore, the integration of variables from the clinical and radiomics models resulted in the establishment of a integrated model. The results of the radiomics model, clinical model, and combined model are presented in **Table 4**.

**Table 4:** The performance of the prediction models.

Models	C_index^*^		ICI		E50		AIC	
	Train	Test	Train	Test	Train	Test	Train	Test
Clinic model	0.676(0.633-0.721)	0.696(0.566-0.829)	0.015	0.071	0.014	0.056	10.987	9.498
Radiomics model	0.736(0.695-0.778)	0.708(0.586-0.794)	0.059	0.051	0.055	0.059	26.941	33.328
Combined model	0.791(0.713-0.851)	0.776(0.654-0.894)	0.014	0.069	0.012	0.04	12.864	11.492

^*^C_index, concordance index; ICI, integrated calibration index; E50, expected prediction error for a given time; AIC, akaike information criterion.

The results showed that the integrated model exhibited superior predictive capabilities in comparison to individual models. In the training set, the C-index was 0.791 [95% confidence interval (CI): 0.713-0.853], ICI was 0.015, E50 was 0.014, and AIC was 10.987. In the testing set, C-index was 0.776 (95% CI: 0.654–0.894), ICI was 0.069, E50 was 0.04, and AIC was 11.492 (**Table 2**).

### Model interpretability

**Figure 5:**
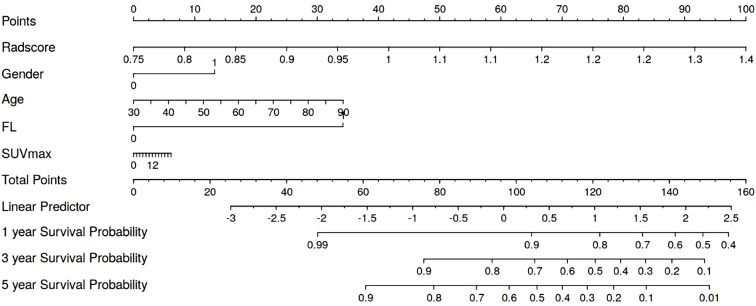
Combined model nomogram.

**Figure 6:**
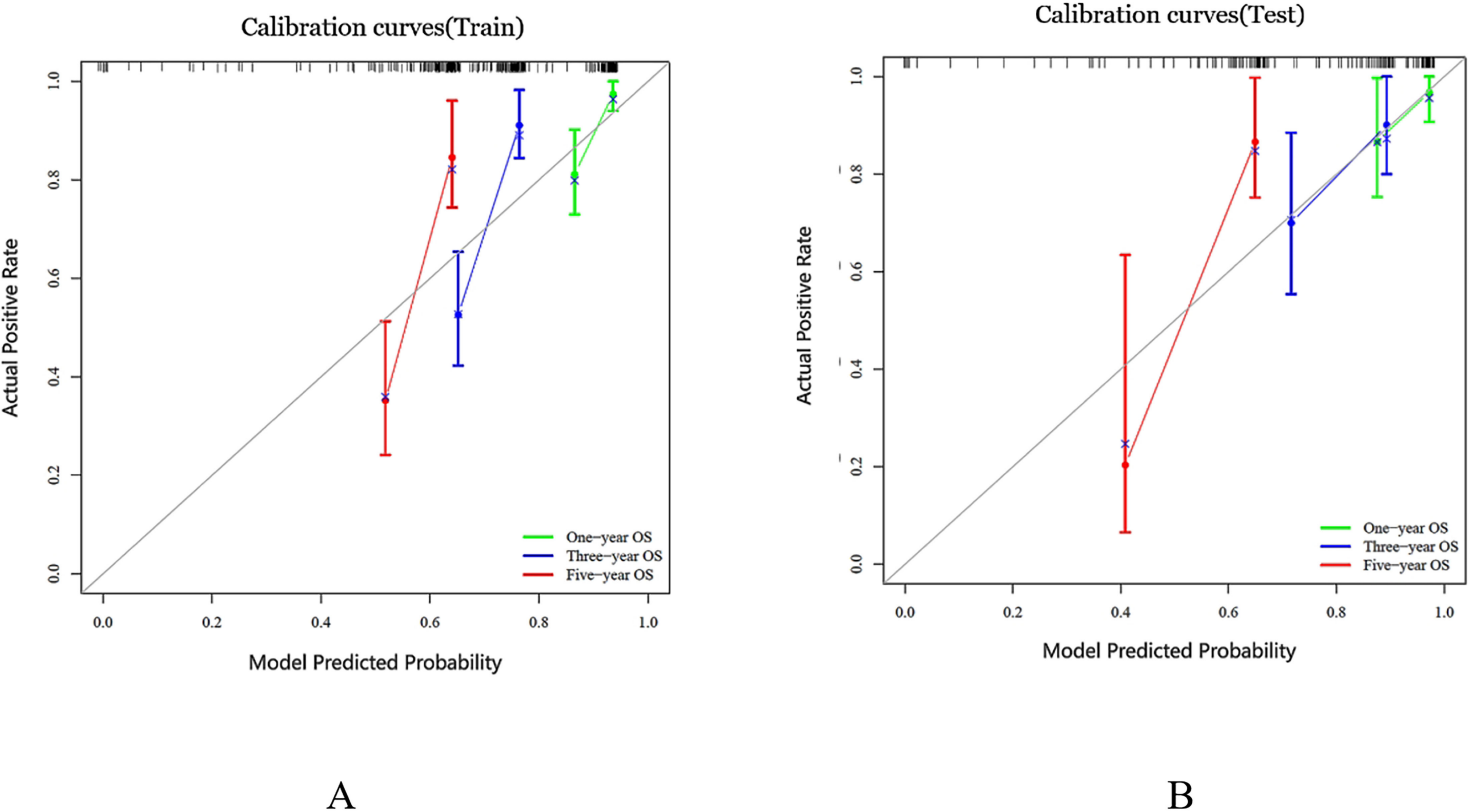
Calibration curves were generated for the prediction model in both the training and test cohorts, represented as **(A, B)** respectively. The ideal prediction is depicted by the black solid line, while the red, blue and green lines showcase the model’s predictive ability. The closer these lines fit to the dashed line, the higher the model’s accuracy in making predictions.

We generated a nomogram to predict the probability of long-term outcomes using the multi-clinical feature set (**Figure 5**). A total score was calculated by summing the scores of five factors: Age, SUVmax, FL, Gender, and CT radiomics score. The corresponding 1-year, 3-year, and 5-year survival rates were associated with the total score. Besides, calibration curves of the integrated model in both the training and validation set are illustrated in **Figure 6**. In this depiction, the diagonal line symbolized the predictive accuracy of an ‘ideal model’, whereas the curve represented the predictive performance of the integrated model. The proximity of the curve to the diagonal line in the graph suggests a higher level of calibration in the model. The findings presented in **Figure 6** illustrate a strong alignment between predicted values and observed outcomes in both the training and validation datasets, thus confirming the model’s dependability and precision in forecasting long-term results.

### Survival analysis

**Figure 7:**
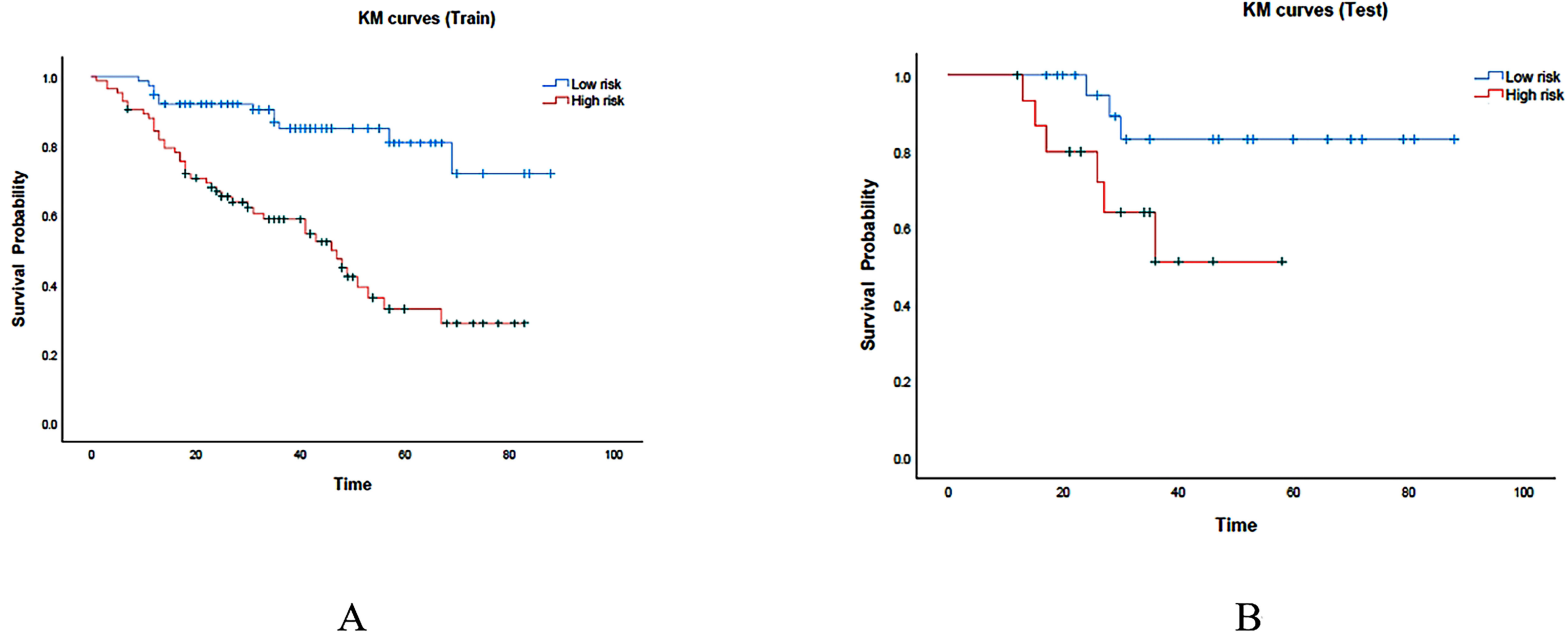
KM curves were generated for the prediction model in both the training and test cohorts, represented as **(A, B)** respectively. The red region represents the high-risk group, while the cyan region represents the low-risk group.

At the end of follow-up, it was determined that out of 199 patients, 64 (32%) had died, while 135 (68%) had survived. The median survival duration was 35 months. The median value of risk prediction from the combined model served as a threshold to categorize patients into high-risk and low-risk groups. In the training set, the high-risk group had a median survival time of 29.0 months (95% CI: 28.5-29.5), whereas the low-risk group had a median survival time of 41.0 months (95% CI: 40.5-41.5).The survival outcomes of the low-risk group significantly exceeded those of the high-risk group (P < 0.001) (**Figure 7A**). In the testing set, the high-risk group showed a median survival time of 30.0 months (95% CI: 28.2-31.8), whereas the low-risk group exhibited a median survival time of 40.5 months (95% CI: 38.1-42.9). The survival outcomes in the low-risk group were significantly better than those in the high-risk group (P = 0.035). Kaplan-Meier (KM) curves for both high-risk and low-risk groups of patients are shown in **Figure 7B**. The ROC curves for survival prediction are shown in the **Supplementary Figure S1**.

## Discussion

In this retrospective multicenter study, we developed a Cox regression model based on CT radiomics features and multi-clinical factors to predict long-term prognosis in patients diagnosed with MM. Through the implementation of an independent data validation model, the integrated model exhibited notable discrimination and calibration capabilities. Subsequently, the model facilitated the stratification of MM patients into high-risk and low-risk subgroup, showcasing significant disparities in OS. Moreover, the integrated model nomogram was developed to offer clinicians with a more intuitive and concise prognostic tool for guiding personalized treatment strategies for patients with MM.

Currently, clinical evaluations predominantly utilize blood-based laboratory parameters to assess effectiveness and prognosis in patients. Nevertheless, this method is associated with limitations such as invasiveness and susceptibility to sampling errors due to the restricted sample size. Bone marrow punctures, a frequently employed technique, may fail to capture regions with the greatest disease burden, thereby restricting its capacity to comprehensively represent the diversity of MM (21). Prior studies have explored the correlation between imaging data and MM survival (22, 23). Relevant findings suggested that Apparent Diffusion Coefficient (ADC) could assess of tumor burden in patients. However, bone marrow ADC measurements are influenced by various factors including the bone marrow cell count, cell morphology, intracellular nuclear-cytoplasmic ratio, size of extracellular spaces, and cell membrane adhesion (24). PET/CT serves as an essential tool for MM assessment, demonstrating that factors such as the count of focal lesions, metabolic activity, volume of highly metabolic lesions, and extramedullary lesions significantly influenced survival. Our recent research suggested that radiomics models could be used to differentiate newly diagnosed myeloma lesions (25, 26). In this study, we developed an integrated model based on radiomics features derived from PET/CT and proceeded to evaluate the prognostic performance of this model.

The application of 18F-FDG PET/CT in the diagnostic and therapeutic evaluation protocols of MM has attained a high level of evidence, making it a valuable method for clinical decision-making, prognostic assessment, and treatment efficacy evaluation. However, only a few studies have determined the prognostic value of radiomic features in MM. Yang et al. confirmed a significant correlation between bone marrow MRI radiomic features and OS in MM patients using Cox regression models. Furthermore, the predictive performance of radiomic features based on radiomics is far superior to traditional clinical models (27). Currently, there is a limited number of studies that incorporate CT-based radiomic features, in conjunction with clinical parameters, to predict the five-year survival rate of patients with MM. This study has identified, for the first time, features extracted from PET/CT that have demonstrated utility in reflecting the biological behavior of MM. In this study, a total of 11 radiomics features extracted from CT scans were employed to construct both clinical and radiomics models, ultimately amalgamating into a combined model for predicting survival outcomes. Univariate and multivariate analyses revealed that SUVmax, Age, FL, and Gender were independent risk factors for predicting the survival of patients with MM. Our study found that the specklenoise_ngtdm_Strength feature has the highest weight ranking and is of significant importance for prognostic assessment. This feature is primarily used to quantify the difference between the voxel gray-level value and the average gray-level value of its neighborhood, reflecting the heterogeneity of the tumor image. A high value may indicate more texture changes, which are associated with higher malignancy or poor prognosis. Previous studies have also shown that in pancreatic ductal adenocarcinoma, high-T1WI_NGTDM_Strength is significantly correlated with poor prognosis (28). In addition, our research results indicate that wavelet-transform-based features demonstrate significant predictive value in the prognostic analysis of MM. This may be because wavelet features can capture the internal heterogeneity of tumors through multi-scale and multi-directional transformations of the images (29).

Our research findings indicate that both the SUVmax value and FL≧ 3 are independent adverse prognostic indicators for individuals with MM, which is consistent with previous research findings (30, 31). Furthermore, our investigation underscored age and gender as additional independent risk factors associated with an unfavorable prognosis in patients with MM. This observation could potentially be explained by a higher incidence of MM in men compared to women, suggesting gender-related disparities in disease susceptibility. Other factors known to affect MM prognosis include RISS, elevated LDH levels, hypercalcemia, renal insufficiency, and treatment group baseline TLG, and so on. However, in this study, due to limited data and selection bias, these factors may not have reached significance. Compared to other factors identified in this report, these factors may also have lesser prognostic significance.

Based on these clinical factors, a clinical model was constructed to predict efficacy. However, the clinical model only showed moderate predictive potential. Integrating clinical predictive factors with radiomics features, a clinical-radiomics combined model was constructed to exhibit a more robust model, outperforming both the individual clinical model and radiomics mode. This integrated model effectively categorized patients into high-risk and low-risk groups with significantly different OS. Radiomics features obtained from CT images of this study play an important role in improving model performance. After feature selection and dimensionality reduction, the retained features include first-order histogram features and texture features. The first-order histogram features include mean, median, minimum, maximum, and standard deviation. These parameters reflect the tumor density on plain scan. Texture features refer to the grayscale variations in images, including gray level co-occurrence matrix, autoregressive texture model, and wavelet transform, which can effectively reflect the heterogeneity of the internal structure of tumors. For high-risk group patients, the model guided clinicians in implementing appropriate treatments promptly. It is recommended to implement standardized treatment plans as early as possible within patient tolerance, and, if necessary, combine advanced therapy to improve prognosis. Moreover, to aid clinical-radiomics decision-making, we visualized this integrated model through a nomogram. This graphical representation streamlined the process for clinicians, swiftly providing predictive insights and enhancing the practical utility of the integrated model in real-time patient management.

There are certain limitations in this study. First, this retrospective study collected images from two centers resulting in a relatively small sample size and potential selection bias. In this study, PET/CT scans were sourced from two different centers, which may have introduced some variability. Although we normalized the images during the data pre-processing phase, including intensity normalization, this process may not have completely eliminated the differences between centers. To further mitigate this variability, we plan to introduce stricter image protocol standardization measures in future studies, such as employing techniques like ComBat tuning to reduce scanner-to-scanner variability. Additionally, this study relied solely on internal validation cohorts and lacked external independent validation cohorts. This limitation restricts the external validity of the model in different patient populations, clinical workflows, and imaging systems. Future studies should incorporate external validation cohorts to further assess the stability and applicability of the model. We plan to collaborate with more medical centers to obtain a broader dataset for comprehensive external validation. Secondly, variations in treatment plans, each with distinct working mechanisms, were administered to patients, potentially influencing therapeutic outcomes and prognosis. In this study, we primarily utilized LASSO regression for feature selection, which is highly effective for dimensionality reduction and feature selection. However, LASSO regression may not fully capture nonlinear relationships between features. To more comprehensively assess feature importance in future studies, we may consider alternative methods such as recursive feature elimination (RFE) or random forest-based approaches. These methods are better suited to capturing complex, especially nonlinear, relationships between features. Additionally, we plan to explore ensemble learning techniques, such as XGBoost or LightGBM, which not only handle nonlinear relationships effectively but also offer enhanced feature selection capabilities. Nevertheless, LASSO regression remains a reasonable choice in this study due to its interpretability, computational efficiency, and robustness in high-dimensional data.

Despite the use of LASSO regression for feature selection, the risk of overfitting persisted due to the relatively small sample size (n = 199) and the large number of radiomics features extracted (over 2000). In particular, the model’s generalization ability may be compromised when the number of features approaches or exceeds the number of events (in this study, the number of deaths was 64). To enhance model robustness and reduce the risk of overfitting, future studies may employ Bootstrap resampling or more stringent cross-validation strategies, such as repeated randomization cross-validation. Additionally, exploring other regularization methods, such as Elastic Net, may help better balance feature selection and model complexity. Another limitation was the analysis solely focused on the lesion with the highest uptake, neglecting other potentially relevant lesions. The radiomics analysis in this study was based solely on a single maximal lesion per patient, which may overlook the multifocal heterogeneity characteristic of multiple myeloma (MM). This approach may limit the model’s ability to capture the full extent of disease burden. Given that heterogeneity is a key feature of MM, multifocal analysis could provide a more comprehensive understanding of the disease. Therefore, future studies should consider developing segmentation algorithms capable of analyzing all lesions throughout the body, rather than focusing exclusively on the largest single lesion. By examining the radiomic characteristics of multiple lesions, the heterogeneity of the disease can be more fully reflected, potentially enhancing the model’s capacity to capture the overall disease burden. Additionally, the manual delineation of ROI was labor-intensive and time-consuming. In terms of lesion segmentation, although we assessed the consistency of segmentation results between two radiologists in this study and retained only features with good reproducibility (ICC > 0.70), the inherent subjectivity of manual segmentation remains a potential limitation. Variability in manual segmentation can influence the stability and reproducibility of radiomics signatures, which in turn may impact model performance. To address this issue, future research could consider incorporating automated or semi-automated segmentation algorithms to minimize the subjectivity associated with manual segmentation. Additionally, the development of detailed segmentation protocols and operational guidelines would be beneficial in standardizing the segmentation process. Conducting multi-reader studies to evaluate the consistency and reliability of segmentation results is also an important direction for future research. These approaches could collectively enhance the robustness and reproducibility of radiomics analyses. To address these limitations in future research, advancements in artificial intelligence technology are anticipated to facilitate automatic ROI segmentation, streamlining the process. This development would enable comprehensive fusion analysis encompassing all lesions, enhancing the thoroughness and accuracy of subsequent evaluations. Cytogenetic information holds significant prognostic value for multiple myeloma. This is a limitation we intend to address in future research, as high-risk cytogenetic features are crucial for improving prognostic accuracy.

## Conclusions

Our study illustrated the potential of an integrated model that combined laboratory examination indicators with PET/CT radiomics features in predicting OS for patients with MM. This integrated model exhibited superior predictive capability compared to individual clinical and radiomics models, showcasing its effectiveness in both training and testing groups. The implementation of this integrated model could help tailor individualized treatment strategies and accurately predict prognosis for patients diagnosed with MM.

## Data Availability

The raw data supporting the conclusions of this article will be made available by the authors, without undue reservation.

## Supplementary material

Supplementary Figure 1 ROC curves for survival prediction.
