# Supplementary figures and images for: Prognostic significance of FDG-PET/CT based radiomics analysis in newly-diagnosed multiple myeloma: a comparative study with clinical assessment
Source: Front Oncol. 2025 Sep 3;15:1486495. doi: 10.3389/fonc.2025.1486495 (PMC12441919; doi:10.3389/fonc.2025.1486495)

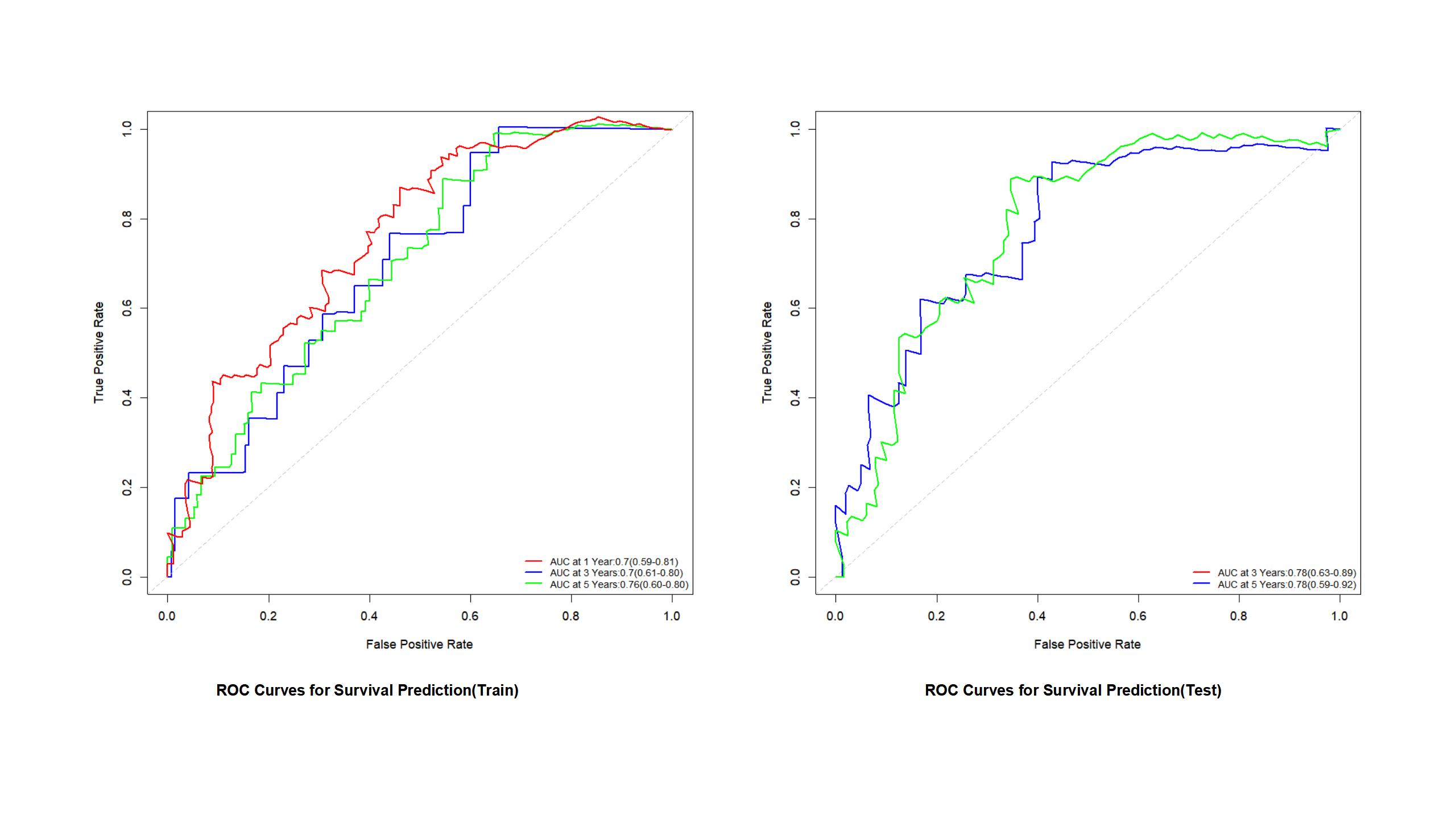

Supplement: Supplementary Figure 1 — ROC curves for survival prediction. [file Image1.jpeg]
